# In This Issue

**Published:** 2000

## Introduction

The use of animals to model humans has long been an integral part of medical and scientific research into human functions and conditions. The development of animal models for alcoholism began in the 1940s. Since that time, a variety of animals have been used to model different drinking behaviors and to study how alcohol damages different bodily organs. Animal models also have helped scientists to analyze the changes in brain chemistry that occur when alcohol is consumed. Perhaps most promising, genetically altered animal models are proving to be invaluable in the search for genes that may be involved in the development of alcoholism. Indeed, a key advantage of animal research, especially research into complex disorders such as alcoholism, is that it enables scientists to simplify complex behaviors by producing fundamental models that are relevant to the human situation.

In developing our issue of *Alcohol Research & Health* on “Animal Models,” we quickly found that the existing literature on this topic far surpassed our page limit for a single issue. To include every topic, only a cursory mention would be possible. Though it breaks with tradition, we felt it would serve our readers better to cover each topic in depth and to dedicate two full issues to the study of animal models in alcohol research.

This first issue gives a broad perspective on the use of animals for investigating the behavioral and physiological effects of alcohol, with only a brief mention of the burgeoning field of genetics research. The second issue will examine in greater detail the use of animals in the search for the genes involved in alcoholism. Together these two issues will provide an excellent review of how animals are helping scientists to better understand the complexities of alcoholism and the effects of alcohol on the human mind and body.

Enoch Gordis, M.D., Director, National Institute on Alcohol Abuse and Alcoholism, National Institutes of Health

## ANIMAL MODELS IN ALCOHOL RESEARCH

For both ethical and scientific reasons, animal models are critical to the study of alcohol use and dependence. Animal models allow researchers to use techniques that cannot be used in humans, such as genetic engineering, and to conduct carefully controlled experiments. Drs. Boris Tabakoff and Paula L. Hoffman introduce this topic both by discussing how animal models are being used in alcohol research and by describing the various animal models that have been developed to study alcohol-seeking behavior, alcohol-related organ damage, tolerance to alcohol, physical dependence on alcohol, and the genetic determinants of alcoholism. (pp. 77–84)

## MODELS OF ALCOHOL’S MOTIVATIONAL EFFECTS

What motivates a person to drink or not to drink? According to Drs. Christopher L. Cunningham, Tara L. Fidler, and Katherine G. Hill, a person may be motivated to continue or increase drinking by alcohol’s positive effects on mood, or he or she may be motivated to discontinue or decrease drinking by alcohol’s negative effects, such as hangover. The authors describe various types of animal models that have been developed to study alcohol’s motivational effects, with a focus on models that directly measure seeking or avoidance of alcohol. They also review the key research findings for each model and discuss issues related to the interpretation of those models. (pp. 85–92)

## MODELING ALCOHOL’S EFFECTS ON ORGANS IN ANIMAL MODELS

Animal models serve not only to investigate the mechanisms underlying the development of alcoholism but also to study alcohol’s effects on various organ systems and on the developing fetus, report Drs. Biddanda C. Ponnappa and Emanuel Rubin. For example, researchers have used animal models (primarily rodents) to analyze alcohol’s detrimental effects on the liver and to identify the mechanisms contributing to alcohol-related liver damage. Other models have provided insights into the mechanisms leading to alcoholic heart disease and have helped researchers explore the consequences of prenatal alcohol exposure. Drs. Ponnappa and Rubin caution, however, that these models cannot reflect all consequences of human alcohol use. For example, in contrast to the human disease, alcohol-related organ damage in animals generally is fully reversible once alcohol exposure is terminated. (pp. 93–104)

## MODELS OF ALCOHOL WITHDRAWAL

Sudden cessation of alcohol consumption after prolonged excessive drinking frequently results in the development of withdrawal symptoms. Dr. Howard C. Becker reviews various animal models that have allowed researchers to study risk factors, underlying mechanisms, and manifestations of alcohol withdrawal. These studies have been conducted both in intact animals and in isolated cells or tissue slices. Using these approaches, researchers have demonstrated a genetic predisposition to the development of withdrawal symptoms. Moreover, animal models have helped investigators identify and analyze numerous physiological and behavioral measures of withdrawal, such as convulsions and anxiety. (pp. 105–113)

## MODELING ADOLESCENT DEVELOPMENT AND ALCOHOL USE IN ANIMALS

Though certain characteristics found in human adolescents are clearly unique, there are other key characteristics of this developmental stage that are common across a number of species. As reported by Dr. Linda Spear, animal models offer researchers unique insight into the effects of alcohol on the adolescent. This age period is particularly important for study, because this is the time during which many people first experiment with alcohol. It is possible that features of the adolescent brain may in fact predispose a youngster to behave in ways that place him or her at particular risk for experimenting with alcohol or other drugs. In addition to behavioral changes, a number of important physiological alterations occur during adolescence. For example, two brain regions that have been implicated in mediating the effects of alcohol and other drugs of abuse have been found to undergo marked alterations during adolescence. (pp. 115–123)

## RECENT ANIMAL MODELS OF ALCOHOLISM

In general, the animal models used in the study of alcoholism are based on how that particular model reacts to alcohol (e.g., preferring to drink alcohol or not). However, as Dr. Rainer Spanagel notes, these preference models may not be appropriate to investigate certain aspects of human alcoholism, such as craving, relapse, or loss of control over drinking. Dr. Spanagel presents three animal models developed in recent years that allow researchers to investigate additional aspects of alcohol dependence and which have been validated pharmacologically. These models include the reinstatement model, which mimics craving; the alcohol deprivation model, which can reflect relapse; and the point-of-no-return model, which allows assessment of the loss of control over drinking. (pp. 124–131)

## AN ECONOMIC APPROACH TO ANIMAL MODELS

A number of animal models exist to mimic human alcohol consumption, yet none of these models can exactly match the way in which a person drinks. Dr. Gene M. Heyman, show s how the principles of economics are now being pressed into service to better predict human alcohol-related behavior. Using concepts such as supply and demand, pricing and income, Dr. Heyman applied economic methods to study rats in a variety of settings. The study found that rats continued to drink alcohol despite as much as a threefold increase in “price.” It also found that food intake during such price increases declined. Dr. Heyman points out that this same type of behavior is often seen in humans addicted to alcohol, supporting the theory that economics can, in fact, help to create an animal model capable of predicting human alcohol-related behavior. (pp. 132–139)

